# A method for reclassifying cause of death in cases categorized as “event of undetermined intent”

**DOI:** 10.1186/s12963-015-0048-y

**Published:** 2015-09-02

**Authors:** Evgeny Andreev, Vladimir M. Shkolnikov, William Alex Pridemore, Svetlana Yu. Nikitina

**Affiliations:** Center for Demographic Research, New Economic School, Nakhimovskii Prospekt 47, 117418 Moscow, Russia; Laboratory of Demographic Data, Max Planck Institute for Demographic Research, Konrad-Zuse-Strasse 1, Rostock, 18057 Germany; University at Albany – State University of New York, School of Criminal Justice, 135 Western Avenue, Draper Hall 219, Albany, NY 12222 USA; Department for population and healthcare statistics, Federal State Statistics Service, 39, Miasnitskaya St., bldg. 1, 107450 Moscow, Russia

**Keywords:** Research methods, Missing data, External causes of death, Events of undetermined intent, ICD codes, Russia

## Abstract

**Background:**

We present a method for reclassifying external causes of death categorized as “event of undetermined intent” (EUIs) into non-transport accidents, suicides, or homicides. In nations like Russia and the UK the absolute number of EUIs is large, the EUI death rate is high, or EUIs comprise a non-trivial proportion of all deaths due to external causes. Overuse of this category may result in (1) substantially underestimating the mortality rate of deaths due to specific external causes and (2) threats to the validity of studies of the patterns and causes of external deaths and of evaluations of the impact of interventions meant to reduce them.

**Methods:**

We employ available characteristics about the deceased and the event to estimate the most likely cause of death using multinomial logistic regression. We use the set of known non-transport accidents, suicides, and homicides to calculate an mlogit-based linear score and an estimated classification probability (ECP). This ECP is applied to EUIs, with varying levels of minimal classification probability. We also present an optional second step that employs a population-level adjustment to reclassify deaths that remain undetermined (the proportion of which varies based on the minimal classification probability). We illustrate our method by applying it to Russia. Between 2000 and 2011, 521,000 Russian deaths (15 % percent of all deaths from external causes) were categorized as EUIs. We used data from anonymized micro-data on the ~3 million deaths from external causes. Our reclassification model used 10 decedent and event characteristics from the computerized death records.

**Results:**

Results show that during this period about 14 % of non-transport accidents, 13 % of suicides, and 33 % of homicides were officially categorized as EUIs. Our findings also suggest that 2011 levels of non-transport accidents and suicides would have been about 24 % higher and of homicide about 82 % higher than that reported by official vital statistics data.

**Conclusions:**

Overuse of the external cause of death classification “event of undetermined intent” may indicate questionable quality of mortality data on external causes of death. This can have wide-ranging implications for families, medical professionals, the justice system, researchers, and policymakers. With our classification probability set as equal to or higher than 0.75, we were able to reclassify about two-thirds of EUI deaths in our sample. Our optional additional step allowed us to redistribute the remaining unclassified EUIs. Our method can be applied to data from any nation or sub-national population in which the EUI category is employed.

## Background

In this paper we present a method for reclassifying external causes of death categorized as “event of undetermined intent” (EUIs). As we show in our study, the probability of a transport accident death being classified as an EUI is very low, thus EUIs caused by external injuries are necessarily due to non-transport accidents, suicides, or homicides. In theory, not enough information exists on EUIs for medical examiners to determine cause of death, though in some cases—especially with homicides and suicides—this category may be used purposely to register the death in this ill-defined category instead of due to a definite or likely violent cause [1–9]. In many industrialized nations use of the EUI category is rare. In some nations, however, the raw number of EUIs is large, the EUI death rate is high, or EUIs comprise a non-trivial proportion of all deaths due to external causes.

Overuse of the EUI category results in meaningful limitations. First, the mortality rate due to non-transport accidents, suicides, or homicides may be substantially underestimated if EUIs are ignored. This is especially problematic if the EUI category is purposely employed to artificially under-enumerate homicide or suicide deaths. As such, use of the EUI category may be considered a proxy for the quality of mortality data on external causes of death [10, 11]. Second, at both the individual and population levels, overuse of the EUI category threatens the validity of studies of the patterns, causes, and consequences of non-transport accidents, suicides, and homicides, and of evaluations of the impact of interventions meant to reduce these types of mortality.

We propose a two-stage method for reclassifying externally caused EUIs as non-transport accidents, suicides, or homicides. After the first stage, a sizeable proportion of EUIs may remain unclassified when we set a higher level of reliability for reclassification. Thus, we add a second optional stage in which we show how reclassification of the entire set of EUI deaths may be reached conditional upon an additional assumption. We illustrate our method by applying it to data on nearly 3 million deaths due to external causes in Russia, a nation with generally reliable mortality data, high mortality from external causes, and a large number of deaths due to and a high rate of EUIs.

Substantively, reclassification of EUIs tends to elevate mortality from homicides and non-transport accidents to a greater extent than mortality from suicides. If these estimates are valid, then this changes our view of Russian rates of external causes of death, especially of important social barometers like homicide and suicide rates. Methodologically, our proposed method can be applied to other nations, allowing for a better understanding of (1) estimates of specific external causes of death, (2) the impact of the use of the EUI category on true rates of death due to non-transport accidents, suicide, and homicide, and (3) the impact on these causes of death of social, cultural, and economic factors and of public policy.

### Use of the EUI category in Russia

Between 2000 and 2011, 15 % percent of all deaths from external causes in Russia were categorized as events of undetermined intent. Table 1 shows that Russia is the dubious leader on this indicator among several select industrialized nations. Other industrialized nations with a meaningful proportion of all deaths from external causes placed in this category include the UK (12 %), Poland (10 %), and Sweden (8 %). While the percentage difference between Russia and the UK seems relatively minor, the Russian age-standardized death rate (SDR) for this category is 8.5 times higher than in the UK and 4.7 times higher than in Poland, the nation with the second highest SDR for this category. Therefore, the Russian problem with EUIs is not only the high proportion of all external causes of death placed in this category but the very large number of deaths. Between 2000 and 2011 there were 521,000 EUI deaths, or more than 43,000 deaths annually. This compares to 541 thousand deaths from suicide and 380 thousand deaths from homicide during this period. If these EUIs were classified correctly it likely would substantially increase Russian rates of non-transport accidents, suicide, and homicide.

**Table 1:** Age-standardized death rates per 100,000 residents for several external causes of death and proportion of all external deaths categorized as due to events of undetermined intent (EUI) among select industrialized nations, 2000–2011

Nation	Years	Cause of death (rates per 100,000 residents)					EUI % share of all external causes
		All external causes	Non-Transport accident	Suicide	Homicide	EUI	
Australia	2001–2011	35.5	13.7	10.3	1.1	1.2	3.4
Austria	2002–2011	40.9	14.8	14.0	0.7	1.3	3.3
Belgium	2003–2009	49.5	17.9	17.2	1.3	1.8	3.7
Canada	2000–2009	39.4	15.4	10.8	1.5	1.7	4.4
Czech Republic	2000–2011	54.6	25.3	13.4	1.0	3.5	6.4
Denmark	2000–2011	37.6	16.7	10.5	0.9	2.2	5.9
Finland	2000–2011	67.0	35.3	18.8	2.2	1.6	2.4
France	2000–2009	49.5	21.5	15.8	0.7	0.8	1.5
Germany	2000–2011	30.9	10.2	10.5	0.6	2.2	7.1
Hungary	2000–2011	68.9	28.7	23.6	1.9	1.3	1.9
Italy	2003, 2006–2010	28.2	12.1	5.4	0.9	0.1	0.4
Netherlands	2000–2011	27.2	11.0	8.6	1.0	0.4	1.3
Norway	2000–2011	41.3	22.1	11.1	0.9	0.1	0.2
Poland	2000–2011	60.9	23.2	14.7	1.4	6.0	9.8
Romania	2000–2010	59.1	27.9	11.8	2.8	1.2	2.1
Russia	2000–2011	186.2	82.2	29.1	20.8	28.0	15.1
Spain	2000–2011	30.2	11.7	6.6	0.9	0.2	0.6
Sweden	2000–2010	38.7	16.4	11.8	1.0	3.1	8.0
United Kingdom	2001–2010	28.1	11.9	6.5	0.4	3.3	11.8
United States	2000–2010	54.9	20.0	11.0	6.0	1.6	2.9

Note: We used the European population standard (World Health Organization, *European health for all database*, [http://data.euro.who.int/hfadb/]) and we include in the table data only for nation-years in which the ICD-10 was used. For Russia, information is based on vital statistics data available online at http://www.demogr.nes.ru/index.php/en/demogr_indicat/data_description. For all other nations, information is based on the WHO Mortality Database that is available online at http://www.who.int/healthinfo/mortality_data/en/

**Fig. 1:**
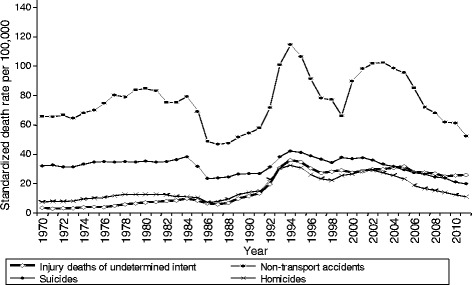
Russian trends in standardized death rates per 100,000 residents for non-transport accidents, suicides, homicides, and external deaths due to events of undetermined intent

Figure 1 shows the Russian SDR due to non-transport accidents, suicides, homicides, and EUIs since 1970. While EUIs generally trend with the other external causes of death, relative to these other causes EUIs (1) rose disproportionately following the collapse of the Soviet Union and (2) have not declined as quickly since the early 2000s. It is important to note that the similarity in trends across many causes of death in Russia, even the change occurring around the collapse of the Soviet Union, is only weakly related to coding practices. Instead, it is mainly explained by the abrupt and painful social, political, and economic changes. This includes the major role played by alcohol, as can be seen with the initiation of Gorbachev’s anti-alcohol campaign in 1985, its weakening in 1988–91, termination in 1991, and subsequent fluctuations in consumption [12–14].

### Our approach to reclassifying death events due to undetermined intent

Our proposed method can be considered a method for imputing missing data. Such methods are often used in demography on census and other population data. Our general approach to reclassifying these deaths is to use other available characteristics about the deceased (e.g., age, sex) and the event (e.g., type of injury, location of death) to estimate the most likely cause of death: non-transport accident, suicide, or homicide. Our approach is based on multinomial logistic regression, which allows one to use these characteristics as explanatory variables to estimate the probability that a case belongs to one of these three causes. In our case, we estimated the classification probability using the characteristics of known non-transport accidents, suicides, and homicides as our training set. We then applied this to the target set: events of undetermined intent. For each death we calculated the most probable category based on its constellation of characteristics. Our estimated classification probabilities (ECP) varied between 0.334 and 0.999, however, and it would make little sense to accept a classification into one of the categories when the ECP is low (e.g., < 0.5). The higher the level of ECP the greater the agreement between deaths from predicted and actual causes of death, but also the higher the number of EUI deaths that cannot be reclassified using the prediction model. At every level of ECP, more EUI deaths were reclassified as homicides and non-transport accidents relative to EUI deaths that were reclassified as suicides. Once we set a minimum limit of ECP to 0.75, it was possible to reclassify about two-thirds of the EUI deaths. Further, if we assume that the probabilities of misclassification of causes of death for the EUI events are the same as the corresponding probabilities for deaths with known causes, it was possible to add an additional optional step and reclassify the entire set of EUI events into one of the three known causes.

## Methods

### Data

Our analyses were based on anonymous micro-data on all deaths from external causes that occurred in Russia between January 1, 2000, and December 31, 2011. These included 1.481 million deaths due to non-transport accidents (ICD-10 codes W00-X59), 541 thousand deaths due to suicide (X60-X84), 379 thousand deaths due to homicide (X85-Y05, Y08, Y09), and 512 thousand deaths due to EUI (Y10-Y34). We excluded from our analysis deaths due to transport accidents (which have a low probability of being classified as an event of undetermined intent; see discussion below in the Sensitivity Analyses section) and a very small number of deaths due to “Neglect and abandonment, and other maltreatment syndromes” (Y06-Y07). Our total number of cases was about 2.913 million.

Each computerized death record includes the following information. (1) Month and year of death registration. (2) A code for the region (analogous to province or state) in which the death was registered, which is usually (but not always) the same as the region of permanent residence of the deceased. (3) Sex. (4) Date of death. (5) Date of birth. (6) Age at death in completed years. (7) Two ICD-10 codes for cause of death. The first ICD code classifies cause according to external cause (e.g., accidental fall or homicide) and the second code denotes the anatomic character of injury (e.g., skull fracture or open wound of thorax). (8) Two aggregated cause of death codes from the abridged Russian cause of death nomenclature corresponding to the ICD-10 codes. We note, however, that our study is based on micro-data from death records, and in these records causes of death are coded by the original ICD-10 items. Thus, we depend on the original ICD-10 coding not the aggregated causes of death used by the Russian statistical agency. (9) Place of death: hospital, outside of a hospital, unknown. (10) The person who issued the death certificate: physician, feldsher (this is a medical worker of an intermediate level between a nurse and a physician), pathologist, or forensic expert. (11) A yes/no indicator of if the deceased was in a state of alcoholic intoxication at the time of death. (12) And a yes/no indicator of if the identity of the deceased was known.

### Methods

#### The multinomial logistic model

Our indirect statistical method for reclassification of EUIs is based on the use of multinomial logistic (mlogit) regression. Beginning with the set of all deaths from the three known causes—non-transport accident, suicide, and homicide—as our training set, we calculated an mlogit-based linear score and a predictor function equal to the estimated classification probability (ECP) that the case in question belongs to one of these three categories. Presuming (for simplicity) that all explanatory variables are dichotomous variables, the multinomial regression model can be expressed as

$$ \Pr \left(n, cause=i\right)=\frac{e^{{\displaystyle \sum_k{B}_{ik}{x}_{ik}^n}}}{{\displaystyle \sum_i{e}^{{\displaystyle \sum_k{B}_{ik}{x}_{ik}^n}}}}. $$

In this equation, causes of death (i.e., outcomes) are numbered *i* = 1, 2, 3. *x*_*ik*_^*n*^ are values of independent dichotomous variables for the case *n*. Index *k* runs across independent variables. *B*_*ik*_ are the respective regression coefficients. One of the three outcomes (say *i* = 3) is considered as a base outcome with *B*_3k_ = 0. Other regression coefficients are estimated by the mlogit procedure according to the maximum likelihood. For every fixed *n*, values of the sum ${\displaystyle \sum_k{B}_i{x}_{ik}^n}$ constitute the corresponding estimated linear scores, and the three values of the prediction function *Pr*(n, cause = *i*) are the estimated probabilities of the three causes of death, with their total equal to 1 (these are the ECP probabilities).

As the number of deaths varies substantially across the three causes, we use weights to eliminate this difference so that the estimation procedure does not give preference according to relative sizes. As a sensitivity check we assessed two regression models with and without weights and compared their results.

To evaluate robustness of regression outcomes and the impacts of errors on the redistribution of the EUIs, we used bootstrapping on the training set to estimate the influence of errors in the regression coefficients *B*_*i*_ on the final result. We generated 250 vectors of coefficients ${\tilde{B}}_i$ using the formula ${\tilde{B}}_i={B}_i+S{E}_i\cdot \gamma$, where *SE*_*i*_ is the standard error of the regression coefficient *B*_*i*_, and *γ* is a random variable that has a standard normal distribution. We applied each vector of coefficients ${\tilde{B}}_i$ for reclassifications of EUIs and examined variation in the results.

Preliminary analyses indicated substantial differences in the results for men and women, thus we conducted separate analyses for each.

#### Independent variables

While a set of independent variables must be informative enough to successfully reclassify the EUI cases into the three causes of death, increasing the number of variables increases the risk of singularities in the Hessian matrix. Therefore, we constructed a variable list such that the Hessian matrix would be non-singular and each variable would be significant at *p* < 0.01 for at least one sex and at least one value of the dependent variable (i.e., cause of death). After a number of experiments, we generated the following list of ten independent variables.

1. Knowledge of identity: A dichotomous variable that informs us if the identity of the person was known and/or the exact date of birth was known to the party registering the death [15].
2. Age group: 0–14, 15–24, 25–34, 35–44, 45–54, 55–64, 65+, and unknown. Although we have the exact age, allowing a continuous range did not improve the accuracy of the model.
3. Year of death: This is especially important due to substantial differences in mortality dynamics over the 2000s. There was an increase in 2000–2002, stabilization and slow decline in 2003–2005, and steeper decline in 2006–2011.
4. Type of day: A dichotomous variable coded 1 if the death occurred on Monday or on a day following a national holiday and 0 if the death occurred on any other day [8]. Any further detail on day of death did not improve the accuracy of the regression model.
5. Season of year: A categorical variable denoting winter (December-February), summer (June-August), and a combined value for spring and autumn. Using the exact month of death did not improve the accuracy of the model.
6. Geographic region: This was based on the eight Federal Districts of Russia, with two exceptions. First, we created one additional region by combining the city of Moscow and its surrounding region (i.e., Moscow Oblast). Second, Stavropol Krai was included as part of the Southern Federal District. Thus, the final number of geographic regions was nine.
7. Urban/rural residence: A dichotomous variable defining whether the death occurred in an urban or rural area.
8. Type of injury: While the list of ICD-10 codes for these injuries includes 195 categories, the Russian national classification contains only 10 aggregate categories. We retained the Russian national classification but added nine additional categories for a total of 19. Table 2 contains a list of our categories, together with the corresponding ICD-10 codes.
9. Presence of alcoholic intoxication at death: A dichotomous variable coded 1 if alcohol intoxication at time of death was acknowledged on the death certificate.
10. Specific location of death: Based on ICD-10 rules [16], the eight places of death were home, residential institution, school or other institution and public administrative area, sports and athletics area, street and highway, trade and service area, other specified places, unspecified place.

**Table 2:** Russian causes of death for classifying deaths by character of injury and their correspondence to items of ICD-10

	Injury	ICD-10 Code
1	Fracture of skull and facial bones	S02
2	Intracranial injury	S06
3	Other injuries to the head	S00, S01, S03-S05, S07-S09
4	Injuries to the neck	S10-S19
5	Open wound of thorax	S21
6	Other injuries to the thorax	S20-S29
7	Injuries to the abdomen, lower back, lumbar spine and pelvis	S30-S39
8	Injuries to the limbs	S40-S99
9	Effects of foreign body entering through natural orifice	T15-T19
10	Burns and corrosions	T20-T32
11	Frostbite	T33-T35
12	Poisoning by narcotics and psychodysleptics [hallucinogens]	T40
13	Toxic effect of alcohol	T51
14	Toxic effect of carbon monoxide	T58
15	Other poisoning by drugs, medicaments and biological substances, toxic effects of substances chiefly nonmedicinal as to source	T36-T65
16	Hypothermia and other effects of reduced temperature	T68, T69
17	Asphyxiation	T71
18	Effects of lightning, drowning and nonfatal submersion, vibration, electric current and other specified effects	T75
19	Other injury, poisoning, and consequences of external causes	T00-T14, T66, T67, T70, T72-T74, T76-T98

Distributions of cases by independent variables is presented in Appendix C. The total number of possible combinations of the independent variables is about 1.120 million. Obviously a majority of them is not provided in the dataset. This set of ten independent variables appeared to be optimal. Adding other explanatory variables either did not reduce the prediction error or led to a singular Hessian matrix.

#### Handling missing values

The input data did not contain missing values. In our mlogit model, the dependent variable (external cause of death) takes three well-defined values: non-transport accident, homicide, and suicide. Explanatory variables may have ill-defined values. For example, age at death may be “unknown” or the anatomic character of the injury may be “unspecified.” However, empirical analysis shows that these ill-defined values provide important information for predicting cause of death. In such cases, therefore, we treated these “unknown” observations as specific values (i.e., unknown) rather than as missing values (coded “.” in statistical packages) and rather than imputing their values.

#### Computations

The mlogit analyses were conducted separately for men and women, though the variable list was the same for both (see the mlogit outputs in the Appendix A). To impute the missing cause of death, we applied the estimated linear scores and corresponding predictor functions to the training set of death records with known causes.

For each case, we estimated classification probabilities of being classified as each of the three death categories—non-transport accident, suicide, homicide—and assigned the case to the cause of death corresponding to the highest probability.

#### Assessing the multinomial logistic model on well-defined cases

Results of the regression based reclassification on the set of deaths with known causes (i.e., non-transport accident, suicide, or homicide) were presented as the distribution matrix D = ‖*d*_*ij*_‖, with *i* and *j* denoting predicted and actual causes of death, respectively (*i,j* = 1,2,3). The nine elements *d*_*ij*_ show a two-dimensional distribution of death cases by predicted and actual causes. *D*_*j*_^*A*^ and *D*_*i*_^*P*^ are the marginal one-dimensional distributions by actual and predicted causes of death, respectively ${D}_j^A={\displaystyle \sum_i{d}_{ij}},{D}_i^P={\displaystyle \sum_j{d}_{ij}}$. Relative error in prediction of the total number of actual cause of events is equal to (*D*_*i*_^*P*^ − *D*_*i*_^*A*^)/*D*_*i*_^*A*^, where (*i* = 1,2,3). The smaller these errors, the closer the model fit of the actual population-level mortality distribution is by cause.

The matrix D was obtained from death records by counting death cases with any of the three estimated ECPs that were greater than or equal to a specific lower limit denoted as ECP_0_. The limit ECP_0_ can be chosen as any value between 0 and 1, with 0 corresponding to full flexibility and 1 to absolute constraint. For every case of death *n*, the candidate cause of death corresponds to the maximum of the three ECPs. However, the final assignment to the respective cause of death depends on the maximum ECP value, such that ECP ≥ ECP_0_. The matrix D corresponding to a specific value of ECP_0_ was denoted ${\mathrm{D}}^{{\mathrm{ECP}}_0}=\left\Vert {d}_{ij}^{{\mathrm{ECP}}_0}\right\Vert$. The relative errors of prediction diminish as the lower limit of ECP_0_ increases. A simple transition from the absolute data $\left\Vert {d}_{ij}^{{\mathrm{ECP}}_0}\right\Vert$ to a relative distribution $\frac{1}{{\displaystyle \sum_{i,j}{d}_{ij}^{{\mathrm{ECP}}_0}}}\left\Vert {d}_{ij}^{{\mathrm{ECP}}_0}\right\Vert$ permits one to compare the latter distributions with respect to the values of the ECP_0_ limits.

#### Constructing the cause-of-death distributions for events of undetermined intent

To reclassify the EUIs, we apply the regression coefficients provided by the multinomial regression model on deaths with known causes. We denote the total numbers of EUIs classified according to the three causes as *U*_*i*_, *i* = 1, 2, 3.

First, we assess *U*_*i*_ for men and women without any restriction on the level of the prediction probabilities (i.e., ECP_0_ = 0). Effectively, in this case the choice of cause *i* is based on the maximal value of ECP without regard to whether its absolute value was high or low. Then, we produce a number of other variants of ${U}_i^{{\mathrm{ECP}}_0}$ corresponding to ECP values that are constrained to be equal to or higher than ECP_0_. For our purposes, we used ECP_0_ values ranging from 0.5 to 0.9.

In our case it was clear that when constraints on ECP values are flexible (e.g., no constraint at all or ECP ≥ 0.5), causes of death can be predicted for all or nearly all EUI cases. Under such conditions, though, a substantial proportion of these predictions could be inaccurate. With stricter constraints on the ECP value (e.g., ECP ≥ 0.8 or ECP ≥ 0.9), however, a relatively high proportion of EUIs can be predicted correctly, but for a substantial proportion of them prediction would be impossible because the maximal (with respect to cause of death *i*) ECP values would not be high enough to fulfill the constraint. The importance of this inevitable balance depends on the quality of diagnostic information contained by the set of independent variables.

Using the results of this reclassification of the set of EUIs we can re-estimate the numbers of deaths and corresponding death rates for non-transport accidents, suicides, and homicides. If we predetermine a higher ECP limit, then some proportion of EUIs remain unclassified. The adjusted number of events belonging to a certain cause of death is the sum of the number of events from this cause among all events with known causes and the number of EUIs reclassified as deaths from the same cause.

It may be that when setting a reasonably high ECP_0_ leaves a relatively high number of cases for which cause of death cannot be predicted at the micro-level by the regression model. However, as an optional second stage we propose a simple procedure for a *population-level* reclassification of all EUIs based on an additional explicit assumption. To do this we return to the classification of cases with known causes of death. The proportion of cases classified by the model as cause *i* actually caused by cause *j* is equal to *P*_*ij*_ = *d*_*ij*_/*D*_*i*_^*P*^. From here it is obvious that ${D}_j^A={\displaystyle \sum_i{P}_{ij}\cdot {D}_j^P}$. Therefore, the proportions *P*_*ij*_ can be considered estimated probabilities for cases classified by the model as cause *i* actually caused by cause *j*. If one assumes that the probabilities of misclassification of causes of death for the EUI events are the same as the corresponding probabilities for deaths with known causes, then the matrix P^*T*^ helps to estimate the population-level distribution of EUIs by causes as ${U}_j^{Adj}={\displaystyle \sum_i{P}_{ij}\cdot {U}_j}$. Again, while this population level redistribution can be of substantial utility, it is an optional step that is not a necessary part of our main redistribution procedure.

## Results

Within the framework of the bootstrap test, we carried out 250 random simulations for each case. In 99.2 % of the cases the predicted cause was the same as the predicted cause based on the original regression coefficients. For males, if the estimated classification probability was equal to or greater than 0.75 then the predicted cause was always the same as the prediction based on the original coefficients. For females this threshold was ECP ≥ 0.77. These tests provided confidence that the identified relationships were not a result of chance.

Table 3 contains the distribution of events by actual and predicted kind of event in the entire dataset and for cases with no lower limit on ECP and with ECP lower limits of 0.5, 0.6, 0.7, 0.75, 0.8, 0.85, and 0.9. In the training set of well-defined death events, the weighted model (for which results are shown in Table 3) correctly predicted the actual causes in 84.5 % (85 % for males, 82 % for females) of cases. It correctly classified 82 % of non-transport accidents, 87 % of suicides, and 92 % of homicides. The unweighted model (not shown in table) correctly classified 86 % of all cases (87 % for males, 85 % for females), including 90 % of non-transport accidents, 85 % of suicides, and 76 % of homicides. So, our choice of the weighted model was justified by the poor performance of the unweighted model on homicide cases. The table shows that the model predicted actual homicides very well. However, the model tended also to over-predict homicide such that when the predicted cause was homicide the actual cause was sometimes different. About 8 % of all cases for males and for females were classified as homicides but were in fact non-transport accidents.

**Table 3:** Distribution of deaths with known causes by actual and predicted cause (per 1000)

	Male					Female				
	Actual cause				Share of agreement	Actual cause				Share of agreement
Predicted cause	Non-transport accidents	Suicide	Homicide	Total		Non-transport accidents	Suicide	Homicide	Total	
All cases										
Non-transport accidents	502	7	2	511	0.98	516	7	5	527	0.98
Suicide	23	208	7	237	0.87	51	153	22	227	0.68
Homicide	84	25	143	252	0.57	79	14	153	246	0.62
Total	609	239	152	1000		645	175	180	1000	
Share of agreement	0.82	0.87	0.94		0.85	0.80	0.88	0.85		0.82
ECP ≥ 0.5										
Non-transport accidents	506	7	2	515	0.98	526	5	3	534	0.98
Suicide	22	209	7	238	0.88	50	157	22	229	0.69
Homicide	82	23	142	247	0.58	71	12	153	237	0.65
Total	610	239	151	1000		647	174	179	1000	
Share of correct predictions	0.83	0.87	0.94		0.86	0.81	0.90	0.86		0.84
ECP ≥ 0.6										
Non-transport accidents	516	7	2	524	0.98	540	4	3	546	0.99
Suicide	22	214	6	242	0.88	45	161	22	228	0.71
Homicide	72	21	140	233	0.60	63	10	153	226	0.68
Total	610	242	148	1000		647	175	178	1000	
Share of correct predictions	0.85	0.89	0.94		0.87	0.83	0.92	0.86		0.85
ECP ≥ 0.7										
Non-transport accidents	539	5	2	545	0.99	567	3	2	573	0.99
Suicide	21	226	7	254	0.89	34	163	22	219	0.74
Homicide	53	16	131	201	0.65	51	7	149	208	0.72
Total	613	248	139	1000		653	173	174	1000	
Share of correct predictions	0.88	0.91	0.94		0.89	0.87	0.94	0.86		0.88
ECP ≥ 0.75										
Non-transport accidents	554	5	2	561	0.99	588	3	2	594	0.99
Suicide	21	234	7	262	0.89	25	160	21	206	0.78
Homicide	42	14	122	177	0.69	46	7	148	200	0.74
Total	617	252	130	1000		659	170	172	1000	
Share of correct predictions	0.90	0.93	0.94		0.91	0.89	0.94	0.86		0.90
ECP ≥ 0.8										
Non-transport accidents	575	4	1	581	0.99	621	3	2	626	0.99
Suicide	20	243	7	270	0.90	17	149	18	184	0.81
Homicide	29	11	109	150	0.73	38	6	145	189	0.77
Total	625	258	117	1000		676	158	166	1000	
Share of correct predictions	0.92	0.94	0.93		0.93	0.92	0.94	0.88		0.92
ECP ≥ 0.85										
Non-transport accidents	607	3	1	612	0.99	696	3	2	701	0.99
Suicide	18	252	6	276	0.91	9	108	12	130	0.84
Homicide	16	8	87	112	0.78	27	6	136	169	0.81
Total	642	263	95	1000		733	117	150	1000	
Share of correct predictions	0.95	0.96	0.92		0.95	0.95	0.93	0.91		0.94
ECP ≥ 0.9										
Non-transport accidents	667	3	1	671	0.99	843	3	2	848	0.99
Suicide	15	254	5	275	0.93	2	34	3	40	0.86
Homicide	5	3	45	54	0.84	11	3	98	112	0.87
Total	687	261	52	1000		857	40	103	1000	
Share of correct predictions	0.97	0.98	0.87		0.97	0.98	0.85	0.95		0.97

Table 4 shows that additional requirements to the minimal ECP level improved this situation, though the problem remained. Indeed, the excess in predicted homicides fell more slowly than the proportion of events of undetermined intent that can be classified. Further investigation of the micro-level data revealed the reason for this phenomenon. It appears that there are nearly homogeneous (in light of the model independent variables) groups of cases that cannot be separated but that contain deaths with different causes. For example, there is a subset of 47 thousand male deaths with registered intracranial injury. The true distribution of events by cause for these cases is 49 % non-transport accidents and 51 % homicides. The problem is that for each case from the first sub-group it is possible to find a case from the second sub-group that looks similarly in light of all other independent variables. However, the weighted and unweighted models classified nearly all these cases as homicides or as non-transport accidents, respectively.

**Table 4:** Errors in estimating the total number of events in the training set by three causes of death, and percentage of reclassified events undetermined intent

	Relative error in prediction of the total number of actual cause of events (percent)			Percent of unclassified events of undetermined intent
	Non-transport accidents	Suicide	Homicide	
No lower limit	−17	4	59	0
ECP ≥ 0.5	−16	5	56	3
ECP ≥ 0.6	−14	5	50	10
ECP ≥ 0.7	−11	6	38	26
ECP ≥ 0.75	−9	6	31	38
ECP ≥ 0.8	−7	6	24	49
ECP ≥ 0.85	−5	6	16	62
ECP ≥ 0.9	−2	5	5	75

Note: Relative error in predicting the total number of actual cause of death is equal (*D*_*i*_^*P*^ − *D*_*i*_^*A*^)/*D*_*i*_^*A*^

Although the weighted model tends to over-predict homicides, this tendency weakens with higher minimum limits on ECP. This implies that the EUIs for a large part of misclassified homicides are relatively low. We can go further to understand why this is happening. First, type of injury is the most informative predictor. Second, some injuries commonly (but not always) correspond to a certain cause of death. The situation “usually but not always” is more characteristic of homicide. For example, an open wound of the thorax in 75 % of events of determined intent corresponds to homicides, and the group “other injury, poisoning and consequences of external causes” corresponds to homicide in 88 % of cases. If we interpret the category “usually but not always” as the share of some kind of events in the range of 66–95 %, then we found that 39 % events of determined intent belong to this category, though 17 % of them are “unusual” events. For non-transport accidents and suicide, the percentage is about 16 % and for homicide it is 33 %. Thus, when increasing the ECP the share of homicides decreases more steeply compared to the two other causes. Once ECP increases, events classified (mostly due to the type of injury) migrate from homicide to the set of unclassified events.

**Fig. 2:**
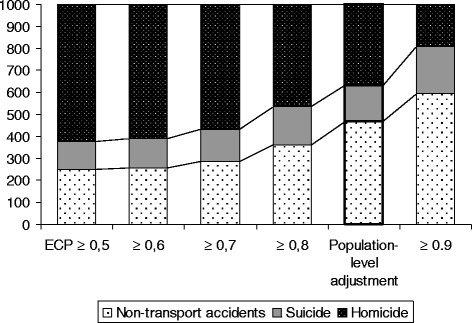
Distributions of the three imputed causes of death (both sexes) depending on constraints on the estimated classification probability, per 1000 cases

Figure 2 illustrates the distribution of reclassified EUI cases by predicted cause of event for different ECP levels. The distribution of the result of population-level adjustment of EUIs by causes is similar to the distribution by causes of EUIs reclassified with ECP ≈ 0.85, providing further evidence of its validity.

Table 5 shows how our model classified the events of undetermined intent. The upper part of the table shows the redistribution of EUIs by causes at different classification probabilities. For men, an increase in the ECP of 0.1 leads on average to a decreased proportion of EUIs reclassified as suicide of 6 per 1000, for non-transport accidents of 18 per 1000, and for homicides of 109 per 1000. For women, the results of reclassification regarding homicides and non-transport accidents looks slightly more resistant, with an increase in the ECP of 0.1 leading to a decrease in their proportion of 91 and 14 per 1000, respectively. The result for suicide demonstrates greater instability than for non-transport accidents. An increase in the ECP of 0.1 leads on average to a decrease in the proportion of EUIs reclassified as suicide of 33 per 1000. The share of suicides declines from 191 per 1000 (with no constraint on ECP) to 6 per 1000 for an ECP ≥ 0.9.

**Table 5:** Results of our reclassifications of the set of events of undetermined intent using multinomial logistic regression, per 1000 cases

	Male				Female			
	Non-transport accidents	Suicides	Homicides	Unclassified	Non-transport accidents	Suicides	Homicides	Unclassified
No constraint on ECP	259	109	632	0	212	191	597	0
ECP ≥ 0.5	254	106	613	27	193	186	550	70
ECP ≥ 0.6	242	104	554	101	175	165	495	164
ECP ≥ 0.7	221	101	419	258	165	133	411	291
ECP > 0.75	209	100	326	366	160	98	296	447
ECP ≥ 0.8	194	96	225	485	154	73	291	482
ECP ≥ 0.85	174	86	130	610	141	37	189	634
ECP ≥ 0.9	156	66	43	736	124	8	64	803
After the population-level adjustment	476	161	363	0	442	167	391	0
After proportional redistribution of unclassified EUIs								
ECP ≥ 0.5	261	109	630	-	208	200	591	-
ECP ≥ 0.6	269	116	616	-	209	197	592	-
ECP ≥ 0.7	298	136	565	-	233	188	580	-
ECP > 0.75	329	158	513	-	289	177	534	-
ECP ≥ 0.8	377	186	437	-	297	141	562	-
ECP ≥ 0.85	446	221	333	-	385	101	515	-
ECP ≥ 0.9	591	250	163	-	629	41	325	-
Known causes	609	239	152	-	645	175	180	-

The lower part of Table 5 has the same meaning as shown in Fig. 2 but shows results for both men and women. One can see that the distribution of the results of the population-level adjustment of EUIs for both men and women by cause is similar to the distribution by cause of EUIs when reclassified with ECP ≈ 0.85. The lower part of Table 5 helps to show that similarity between the actual distribution of known causes of reclassified EUIs increases as the ECP increases. As expected, the proportion of non-transport accidents among all deaths of undetermined intent is lower, and the proportion of homicides is higher, than among deaths of determined intent. The proportion of suicides is about the same.

Table 6 presents the results of calculations based on the distribution of deaths of determined intent after the additional optional population level correction. Similar data by sex are presented in Appendix B. Using the standardized death rates on the right side of the table we can see that at ECP ≥ 0.75, only 6.4 % of deaths under consideration remain unclassified (6.7 % for men and 6.2 % for women). The population level adjustment included the majority of these cases being reclassified as non-transport accidents. A similar situation is observed for both men and women (as seen in Appendix B). For both sexes together, the SDR from suicide is slightly higher than from homicide, but for men this difference is greater (10 per 100,000) and for women the SDR from suicide is lower than from homicide.

**Table 6:** Distribution of deaths by cause according to officially registered vital statistics data and our reclassified figures

	Number of deaths (thousand)					SDR per 100,000				
	Total	Non-transport accidents	Suicides	Homicides	Unclassified	Total	Non-transport accidents	Suicides	Homicides	Unclassified
Actual	2914	1481	541	379	512	160	82.2	29.1	20.7	28.0
ECP ≥ 0.50		1605	605	686	18		89.0	32.5	37.6	1.0
ECP ≥ 0.60		1598	601	657	58		88.6	32.3	35.9	3.2
ECP ≥ 0.70		1589	597	593	136		88.1	32.1	32.4	7.4
ECP ≥ 0.75		1583	593	550	188		87.8	31.9	30.1	10.3
ECP ≥ 0.80		1576	588	502	248		87.4	31.6	27.4	13.6
ECP ≥ 0.85		1567	580	452	315		86.9	31.2	24.7	17.3
ECP ≥ 0.90		1558	569	404	384		86.4	30.6	22.0	21.0
After the population level adjustment		1722	624	568			95.3	33.7	31.0	

Table 7 shows annual SDRs for the years 2000–2011 for (1) deaths officially registered as non-transport accidents, suicides, and homicides, (2) deaths officially registered as being of undetermined intent but that our model classified as non-transport accidents, suicides, or homicides, (3) the adjusted rate (i.e., the sum of these two groups), and (4) the proportion of the adjusted rate accounted for by reclassification of events of undetermined intent. The table shows that the SDRs for deaths that we classified as non-transport accidents or as suicides (but that were officially registered as EUIs) were essentially stable between 2000 and 2011. Due to the decline in the death rate from officially registered suicides, however, the share of all suicides classified as events of undetermined intent grew significantly. The male SDR for deaths officially registered as homicides declined from 47.6 in 2002 to 18.1 in 2011. However, the SDR for deaths that our refined model classified as homicides (but that were officially registered as deaths of undetermined intent) declined at a much slower pace. Therefore, the share of all male homicides that were classified as events of undetermined intent grew. A similar situation is observed for women.

**Table 7:** Annual sex-specific standardized death rates per 100,000 residents for non-transport accidents, suicides, and homicides in Russia according to officially registered vital statistics data and our corrected data after the population level adjustment, 2000–2011

	Male				Female			
	According to vital statistics	Adjusted figures	Initially registered as events of undetermined intent	Proportion of adjusted rate accounted for by reclassification of events of undetermined intent (percent)	According to vital statistics	Adjusted figures	Initially registered as events of undetermined intent	Proportion of adjusted rate accounted for by reclassification of events of undetermined intent (percent)
Non-transport accidents								
2000	152.1	174.9	22.8	13	36.3	40.7	4.4	11
2001	166.6	189.6	23.0	12	40.3	44.8	4.5	10
2002	172.7	196.4	23.7	12	42.3	47.0	4.7	10
2003	174.3	198.2	23.9	12	42.3	47.3	4.9	10
2004	168.1	193.2	25.1	13	40.2	45.4	5.1	11
2005	163.9	190.3	26.4	14	38.7	43.8	5.1	12
2006	145.4	169.7	24.3	14	34.7	39.4	4.7	12
2007	124.0	147.3	23.4	16	28.8	33.5	4.8	14
2008	117.7	140.6	22.9	16	27.3	31.8	4.6	14
2009	106.1	127.5	21.4	17	25.8	30.3	4.5	15
2010	104.7	127.1	22.4	18	25.4	30.1	4.8	16
2011	89.2	111.5	22.2	20	21.8	26.6	4.8	18
Suicide								
2000	68.8	75.8	7.0	9	10.4	12.1	1.7	14
2001	69.8	77.4	7.6	10	10.5	12.2	1.7	14
2002	66.8	74.5	7.6	10	10.4	12.1	1.7	14
2003	62.4	70.1	7.8	11	9.5	11.3	1.8	16
2004	59.2	67.3	8.1	12	9.3	11.1	1.8	16
2005	55.1	63.8	8.7	14	8.5	10.3	1.9	18
2006	50.7	58.3	7.6	13	8.1	9.8	1.7	18
2007	48.4	56.5	8.1	14	8.3	10.1	1.8	18
2008	44.9	52.8	7.9	15	7.9	9.7	1.8	19
2009	44.1	52.0	7.9	15	7.4	9.3	1.9	20
2010	39.3	47.4	8.1	17	6.6	8.4	1.8	22
2011	36.5	44.9	8.4	19	6.4	8.2	1.8	22
Homicide								
2000	42.4	60.7	18.3	30	12.4	16.8	4.4	26
2001	45.5	65.0	19.5	30	13.1	17.7	4.6	26
2002	47.6	68.4	20.8	30	13.0	17.8	4.8	27
2003	44.4	64.8	20.5	32	12.5	17.3	4.8	28
2004	41.8	62.7	20.8	33	11.6	16.6	4.9	30
2005	37.8	59.0	21.2	36	10.6	15.5	4.8	31
2006	30.6	48.7	18.1	37	8.6	12.7	4.1	32
2007	27.0	44.3	17.3	39	7.4	11.5	4.1	36
2008	25.3	42.4	17.1	40	6.9	10.7	3.8	36
2009	22.6	37.6	15.0	40	6.4	9.9	3.6	36
2010	19.8	34.6	14.8	43	5.7	9.2	3.6	39
2011	18.1	33.3	15.2	46	4.9	8.5	3.7	43

### Sensitivity analyses

First, it is possible that our exclusion of transport accidents when reclassifying EUIs biases our results. In theory, some transport accidents—especially deaths due to “Falling, lying or running in front of or into moving object. Undetermined intent.” (Y31) and “Crashing of motor vehicle. Undetermined intent” (Y32)—may be recorded as EUIs. However, these deaths make up only 1.2 % of all EUIs and 1.3 % of all transport accidents. It also would be difficult to include transport accidents in our general reclassification model due to peculiar values on some important explanatory variables. For example, “Place of death” operates very differently in this context and is not comparable with that for deaths due to other external causes. Nevertheless, we executed an additional mlogit model to distinguish between deaths due to transport accidents from those due to the combined group of non-transport accidents, suicides, and homicides. The model identifies transport accidents with a probability of error < .01. Application of the model score to EUI deaths shows that only 1.4 % can be classified as transport accidents (respective percentages for Y31 and Y32 are 1.3 and 3.6 %), while these deaths show much higher probabilities of being homicides or suicides. Results are shown in Appendix D, and they suggest that in Russia transport accidents comprise a distinct group and that they have the potential to produce only a very minor impact on the distribution of EUIs and on the final distribution of external causes of death. Our decision to exclude transport accidents from our reclassification is also supported by information gained from the processing of such cases and by prior research. For example, in Russia nearly all fatal transport accidents are rapidly followed by investigations by the road police (a distinct branch of the Russian police force) and by criminal investigators with forensic expertise, which diminishes the chances for recording bias or classification as EUIs for such deaths. Further, although prior studies of the quality of cause of death diagnoses in Russia found that registration of deaths due to transport accidents has some limitations, these are less problematic than for other types of accidents and violent deaths [1, 3, 4]. Most obviously, the percentage of deaths from “other” and “unspecified” transport accidents comprise only 2.6 and 0.1 % of all deaths from transport accidents, respectively, which is much lower than corresponding categories for non-transport accidents, suicides, and homicides. With respect to possible misclassification as EUIs, prior research focused on homicides and suicides but not transport accidents [2, 5–9].

Second, the default method of redistribution is to reattribute deaths within sex- and age-groups proportionately to the numbers of non-transport accidents, suicides, and homicide in it. An important related question is how much value our model provides over this default method. If our model-based results are very similar to the results from this default method of redistribution, then our model provides little added value (which would be an important finding in itself). This default method of redistribution is a reasonable option in the absence of any other information. A similar method is to assume *a priori* that EUIs are hidden suicides [11, 17] or hidden homicides [3] or both (but not hidden non-transport accidents) [18]. Prior studies of Russia, however, provide additional evidence suggesting non-proportional distributions. With natural causes, for example, there are strong reasons for adding ill-defined deaths from senility to the class of circulatory diseases [19, 20]. For EUIs specifically, the evidence suggests possible misclassification of homicides and suicides [1–9]. In spite of this, we are unaware of any studies that used the reclassification method we are proposing. Still, it is important to compare the corrected distribution of external causes based on our model with the default method of redistribution. We did this and our results are shown in Appendix E. The results show that our model-based redistributions differ substantially from the results of the default solution.

Third, our analyses can be used for two distinct applications. One is to estimate the correct cause of death for any particular individual case. Another is to obtain the best estimate of population-level incidence of each type of injury. It is intuitive to employ the estimated probability as we do for the former, but not necessarily intuitive to use a threshold on the estimated classification probability for the latter. Our primary interest is to establish more precise population-level data on external cause mortality (i.e., the second application), which is why after the individual-level reclassification of EUIs with mlogit we make the population-level adjustment on the EUI cases with the low mlogit probabilities. By employing the cutoff points in assigning cause of death our aim is to provide a more reliable basis for the population-level distribution. When we do so, we assume that the solutions with the mlogit probabilities below the cutoff suggest that insufficient information is provided by the explanatory variables. With the help of combinatorics, we know that the probability of getting (for example) a combination of 8 accidents, 1 homicide, and 1 suicide in ten trials is 0.151. It is also possible to interpret the hypothetical mlogit return of (0.8, 0.1, 0.1) as a vector of classification probabilities belonging to three fuzzy sets of deaths. This three-cause proportional sharing-based approach leads to a specific distribution by cause of death. We show the results of this proportional sharing-based redistribution in Appendix E, and again it is substantially different from our model-based distribution. We thank one of our reviewers for this suggestion.

Finally, we considered the possibility of preliminary conformal grouping because in theory it seems attractive to do separate redistributions for a few more homogeneous subgroups of EUIs within the corresponding specific categories of suicide, homicide, and non-transport accidents. Two reasons, however, make it very difficult to build reliable correspondences between EUI subgroups and the subgroups of non-transport accidents, suicides, and homicides. One reason is that prior studies of Russia [1–9] suggest imprecise registration of single item injuries and of violent causes, as well as high numbers of deaths due to “other” and “unspecified” events within subgroups of accidents and within subgroups of suicide and homicide. In particular, reclassification of falls of unknown intent (Y30) into unintentional falls (W00-W19), suicide by jumping (X80), and assault by pushing from high place (Y01) assumes these categories are reliable without false exchanges with other items. Yet we know that such exchanges are probable due to the low quality of single items and that it is better to use more reliable aggregate categories. Further, Y30 may be confused with Y31 and with Y33 and Y34, and items Y33-Y34 (“Other specified or unspecified events. Undetermined intent.”), which can be included in any group, composed 31 % of all EUIs in Russia during the period under study (2000–2011). The second reason is that there is a formal problem due to the presence of “other” and unspecified categories. One does not know, for example, what part of Y33 and Y34 should be assigned to Y30 and what part of X58-X59 should be assigned to W00-W19 before estimating the regression model.

## Discussion

The rate of external causes of death due to events of undetermined intent is extremely high in Russia, about 28 per 100,000 residents between 2000 and 2011. Their proportion of all deaths from external causes accelerated in the years following the collapse of the Soviet Union, and the rate has not declined at the same pace as known external causes of death over the last decade (Fig. 1 above; [1, 7, 14]). However, Russia and other East European nations are not the only countries to experience limitations in classification of external causes of death. Between 2000 and 2010, for example, Table 1 shows that the proportion of all external deaths classified as events of undetermined intent was 15 % in Russia, 12 % in the United Kingdom, 10 % in Poland, 8 % in Sweden, 7 % in Germany, and 6 % in Denmark and the Czech Republic.

This limitation has important practical, scientific, and policy implications. For example, the rate at which the “event of undetermined intent” category is used may provide an indicator of the quality of vital statistics data, at least for external causes of death [1, 7]. There are legitimate reasons—e.g., truly unknown intent, overworked and understaffed coroner’s offices—to use this category. Unfortunately, there are reasons to believe that in some nations at some times this category may be employed to purposely misclassify homicide and suicide deaths [7, 17, 21]. Whether purposely or as an unintended consequence, another implication is that regular use of this category leads to under-enumeration of rates of important social indicators like homicide and suicide. As we show here, this under-enumeration can be substantial, and annual public reports of homicide and suicide rates rarely allude to EUIs as limitations of the reported rate. Another implication is that scholars interested in the structural covariates of homicide and suicide rates seem largely unaware of this category and do not account for it in their analyses, which may threaten the validity of these studies. The validity of individual-level studies of external causes of death may be similarly threatened, as are studies of interventions aimed at reducing deaths due to accident, suicide, or homicide.

The authors of some prior studies of mortality from violence and accidents in Russia suggested what may be hidden behind numerous death events with undetermined intent. Some scholars of mortality in East European nations believed external deaths due to undetermined intent may consist largely of hidden suicides [17]. Others believed the majority of these deaths were murders [21], or at least that a substantial portion of them are murders and that the misclassification in some instances may be purposeful [1, 7].

A recent study by Ivanova et al. [22] made use of comparisons between deaths from known accidents, suicides, homicides, and events of undetermined intent by employing the distributions of the character of injury for deaths within the range of ages 20 to 59. Focusing on the most frequent combination of the type of injury and cause, their study offered a version of EUI redistribution, with a majority of EUIs being assigned either to homicides (34 %) or suicides (27 %).

Our study extends this recent work by bringing to bear a large set of informative micro-data. We were able to model the relationships between the three causes of death (non-transport accident, suicide, and homicide) and ten independent variables, which allowed us to predict the cause of death for EUI cases. The model tended unambiguously to assign most of EUIs to either homicide or to non-transport accidents, with a smaller role of suicide. With ECP ≥ 0.75, 33 % of EUIs were reclassified as homicides, 20 % as non-transport accidents, and 10 % as suicides, with 37 % remaining unclassified.

If one assumes that the probabilities of misclassification of causes of death for the EUIs are the same as the corresponding probabilities for deaths with known causes, the entire set of EUIs would be distributed with 48 % of cases assigned to non-transport accidents, 36 % assigned to homicides, and 16 % assigned to suicides. This result suggests that the proportion of hidden homicides among EUIs was 131 % higher than the corresponding proportion among the injury deaths of determined intent (36 % vs. 16 %). For suicides, these proportions are 16 % vs. 23 %, and for non-transport accidents they are 47 % vs. 62 %. Although we did not find strong support for the hypothesis that the EUI category is used mainly for hiding murder, the redistribution of EUIs does result in a substantial elevation of the official mortality figures for homicide. After the adjustment, the Russian age standardized homicide rate for 2011 is 20.0 per 100,000, which is nearly double the officially recorded value of 11.1 per 100,000. Similarly, the adjusted suicide rate of 24.9 exceeds the official rate of 20.0 by one-quarter.

There are further implications for homicide. According to our imputation, 33 % of all (i.e., officially recorded plus hidden) homicides were initially classified as EUIs (compared to 9 % of all non-transport accident and 5 % of all suicide deaths). Between 2000 and 2011, this proportion increased from 28 to 44 %. This supports the concerns of some scholars [1, 7] about the quality of the Russian homicide data and the validity of the officially registered reduction in homicide mortality in Russia. According to Antonova’s [23] estimates, the actual number of homicides at ages 20–39 years was about 1.5 times higher than that registered by official data, and at ages 40–59 the actual number of homicides was nearly twice as high as the official figure.

Beyond the quality of vital statistics data and their use by scholars, this also may be considered an important signal for police (which record even fewer homicides than the vital statistics), criminal justice, and society as a whole. While there is no doubt many “hidden” homicides are legitimately classified as events of undetermined intent due to lack of biomedical and legal evidence, it is difficult to ignore the likelihood that a non-trivial proportion of them is hidden due to the weaknesses within the system for investigation or other reasons.

It is not uncommon for Russian pathologists to issue a provisional death certificate, which allows for burial but does not contain the precise cause of death. Although it is assumed a qualified certificate will be issued later to be used for vital statistics registration, in practice this does not always happen. In these cases, agencies must depend on the provisional death certificates. Gavrilova et al. [1] hypothesized that the increase in deaths attributed to unknown causes was due to a growing proportion of “Provisional” death certificates. Using data for 2011, we found that 32 % of deaths registered via a provisional death certificate were EUIs compared to 23 % of deaths registered via a final death certificate. Nevertheless, 80 % of all EUIs are based on final death certificates, so it does not appear that categorizing deaths as due to undetermined intent is a function of insufficient time to make an accurate diagnosis.

## Conclusions

Overuse of the external cause of death classification “event of undetermined intent” may indicate questionable quality of mortality data on external causes of death. This can have wide-ranging implications for families, medical professionals, the justice system, researchers, and policymakers. We propose an indirect statistical method for reclassifying these deaths as non-transport accidents, suicides, or homicides, and at the population level we provide a means of further refining the method’s outcomes. With the classification probability set as equal to or higher than 0.75, about two-thirds of EUI deaths can be reclassified. An additional assumption allows us to employ an optional population level computation to redistribute the remaining unclassified EUIs. To illustrate this method we employed Russian mortality data on nearly 3 million deaths due to external causes, a nation where the use of the EUI category is especially troublesome, and our method returned plausible and meaningful results. The method can be applied to data from other nations or sub-national populations in which the EUI category is employed and for which micro-data with additional information are available.

## Appendix A

**Table 8:** Mlogit outputs (The base outcome is the last category *Homicide*)

		Male			Female		
		B	SE	p-value	B	SE	p-value
1.	Non-transport accidents						
1.0.	Intercept	−3.419	0.058	0.000	−3.382	0.157	0.000
1.1.	Knowledge of identity						
1.1.1.	Identified person	0.770	0.020	0.000	0.520	0.039	0.000
1.1.2.	Unidentified person	0 ^(*)^	.	.	0 ^(*)^	.	.
1.2.	Age group						
1.2.1.	0–14	0.940	0.039	0.000	1.183	0.073	0.000
1.2.2.	15–24	−0.248	0.033	0.000	−0.282	0.070	0.000
1.2.3.	25–34	−0.399	0.032	0.000	−0.358	0.069	0.000
1.2.4.	35–44	−0.294	0.031	0.000	−0.298	0.069	0.000
1.2.5.	45–54	−0.050	0.031	0.111	−0.019	0.068	0.780
1.2.6.	55–64	0.248	0.032	0.000	0.173	0.069	0.012
1.2.7.	65 and older	0.618	0.032	0.000	0.586	0.068	0.000
1.2.8.	unknown	0 ^(*)^	.	.	0 ^(*)^	.	.
1.3.	Year of death						
1.3.1.	2000–2002	−0.197	0.008	0.000	−0.278	0.014	0.000
1.3.2.	2003–2005	−0.191	0.008	0.000	−0.249	0.014	0.000
1.3.3.	2006–2011	0 ^(*)^	.	.	0 ^(*)^	.	.
1.4.	Type of day						
1.4.1.	Monday or a day after national holiday	0.002	0.009	0.816	0.003	0.016	0.858
1.4.1.	Other weekdays	0 ^(*)^	.	.	0 ^(*)^	.	.
1.5.	Season of year						
1.5.1.	December-February	−0.369	0.007	0.000	−0.261	0.014	0.000
1.5.2.	June-August	−0.517	0.009	0.000	−0.342	0.016	0.000
1.5.3.	Other months	0 ^(*)^	.	.	0 ^(*)^	.	.
1.6.	Geographic region						
1.6.1.	Central Federal District except Moscow	−0.344	0.013	0.000	−0.467	0.023	0.000
1.6.2.	North West Federal District	−0.467	0.014	0.000	−0.441	0.025	0.000
1.6.3.	South Federal Districtand Stavropol kray	−0.624	0.015	0.000	−0.847	0.028	0.000
1.6.4.	Volga Federal District	−0.681	0.012	0.000	−0.670	0.021	0.000
1.6.5.	Urals Federal District	−0.846	0.014	0.000	−0.798	0.025	0.000
1.6.6.	Siberian Federal District	−1.327	0.013	0.000	−1.208	0.023	0.000
1.6.7.	Far East Federal District	−0.655	0.015	0.000	−0.448	0.029	0.000
1.6.8.	Republics of North Caucasian Federal District	−0.093	0.023	0.000	0.510	0.051	0.000
1.6.9.	Moscow and Moscow region	0 ^(*)^	.	.	0 ^(*)^	.	.
1.7.	Urban/rural residence						
1.7.1.	Rural area	−0.042	0.007	0.000	−0.199	0.014	0.000
1.7.2.	Urban area	0 ^(*)^	.	.	0 ^(*)^	.	.
1.8.	Type of injury						
1.8.1.	Fracture of skull and facial bones	2.692	0.050	0.000	2.882	0.144	0.000
1.8.2.	Intracranial injury	3.049	0.049	0.000	3.134	0.143	0.000
1.8.3.	Other Injuries to the head	3.083	0.049	0.000	3.443	0.142	0.000
1.8.4.	Injuries to the neck	2.118	0.051	0.000	1.856	0.147	0.000
1.8.5.	Open wound of thorax	1.605	0.050	0.000	2.383	0.143	0.000
1.8.6.	Other injuries to the abdomen, lower back, lumbar spine and pelvis	1.869	0.051	0.000	2.610	0.144	0.000
1.8.7.	Injuries to the abdomen, lower back, lumbar spine and pelvis	3.276	0.052	0.000	5.517	0.144	0.000
1.8.8.	Injuries to the limbs	3.332	0.049	0.000	3.972	0.142	0.000
1.8.9.	Effects of foreign body entering through natural orifice	8.407	0.059	0.000	8.395	0.150	0.000
1.8.2.	Burns and corrosions	7.101	0.054	0.000	7.588	0.146	0.000
1.8.3.	Frostbite	9.378	0.128	0.000	10.384	0.334	0.000
1.8.4.	Poisoning by narcotics and psychodysleptics [hallucinogens]	9.266	0.073	0.000	9.556	0.167	0.000
1.8.5.	Toxic effect of carbon monoxide	11.758	0.114	0.000	11.828	0.231	0.000
1.8.6.	Other poisoning by drugs, medicaments and biological substances, toxic effects of substances chiefly nonmedicinal as to source	9.756	0.066	0.000	9.932	0.157	0.000
1.8.7.	Hypothermia and other effects of reduced temperature	10.881	0.084	0.000	11.499	0.196	0.000
1.8.8.	Asphyxiation	4.332	0.049	0.000	3.552	0.142	0.000
1.8.9.	Effects of lightning, drowning and nonfatal submersion, vibration, electric current and other specified effects	8.055	0.056	0.000	7.996	0.149	0.000
1.8.10.	Other injury, poisoning and consequences of external causes	0 ^(*)^	.	.	0 ^(*)^	.	.
1.9.	Presence of alcoholic intoxication at death						
1.9.1.	No	−0.483	0.020	0.000	−0.942	0.037	0.000
1.9.2.	Yes	0 ^(*)^	.	.	0 ^(*)^	.	.
1.10.	Specific location of death						
1.10.1.	Home	−0.349	0.008	0.000	−0.401	0.014	0.000
1.10.2.	Residential institution	−0.617	0.038	0.000	−0.803	0.071	0.000
1.10.3.	School, other institution and public administrative area	0.098	0.025	0.000	0.117	0.051	0.022
1.10.4.	Sports and athletics area	−0.538	0.015	0.000	−0.295	0.032	0.000
1.10.5.	Street and highway	−0.369	0.046	0.000	−0.834	0.105	0.000
1.10.6.	Trade and service area	2.093	0.026	0.000	1.197	0.084	0.000
1.10.7.	Other specified places	0.233	0.012	0.000	−0.039	0.026	0.142
1.10.8.	Unspecified place	0 ^(*)^	.	.	0 ^(*)^	.	.
2.	Suicide						
2.0.	Intercept	−4.510	0.043	0.000	−5.777	0.110	0.000
2.1.	Knowledge of identity						
2.1.1.	Identified person	1.362	0.023	0.000	1.303	0.040	0.000
2.1.2.	Unidentified person	0 ^(*)^	.	.	0 ^(*)^	.	.
2.2.	Age group						
2.2.1.	0–14	−1.689	0.047	0.000	−1.030	0.082	0.000
2.2.2.	15–24	0.229	0.039	0.000	0.409	0.077	0.000
2.2.3.	25–34	−0.051	0.038	0.180	0.299	0.076	0.000
2.2.4.	35–44	−0.111	0.038	0.003	0.393	0.076	0.000
2.2.5.	45–54	−0.012	0.038	0.748	0.551	0.076	0.000
2.2.6.	55–64	0.131	0.038	0.001	0.637	0.076	0.000
2.2.7.	65 and older	0.797	0.039	0.000	1.066	0.075	0.000
2.2.8.	unknown	0 ^(*)^	.	.	0 ^(*)^	.	.
2.3.	Year of death						
2.3.1.	2000–2002	−0.027	0.008	0.001	−0.189	0.013	0.000
2.3.2.	2003–2005	−0.245	0.008	0.000	−0.315	0.013	0.000
2.3.3.	2006–2011	0 ^(*)^	.	.	0 ^(*)^	.	.
2.4.	Type of day						
2.4.1.	Monday or day following a national holiday	0.077	0.009	0.000	0.048	0.015	0.001
2.4.1.	Other weekdays	0 ^(*)^	.	.	0 ^(*)^	.	.
2.5.	Season of year						
2.5.1.	December-February	−0.082	0.008	0.000	−0.205	0.013	0.000
2.5.2.	June-August	−0.231	0.009	0.000	−0.468	0.015	0.000
2.5.3.	Other months	0 ^(*)^	.	.	0 ^(*)^	.	.
2.6.	Geographic region						
2.6.1.	Central Federal District except Moscow	0.067	0.015	0.000	−0.539	0.024	0.000
2.6.2.	North West Federal District	0.160	0.015	0.000	−0.323	0.025	0.000
2.6.3.	South Federal Districtand Stavropol kray	0.131	0.016	0.000	−0.448	0.026	0.000
2.6.4.	Volga Federal District	0.176	0.013	0.000	−0.390	0.022	0.000
2.6.5.	Urals Federal District	0.227	0.015	0.000	−0.255	0.024	0.000
2.6.6.	Siberian Federal District	−0.135	0.014	0.000	−0.425	0.022	0.000
2.6.7.	Far East Federal District	−0.116	0.016	0.000	−0.395	0.028	0.000
2.6.8.	Republics of North Caucasian Federal District	−0.442	0.032	0.000	−0.296	0.061	0.000
2.6.9.	Moscow and Moscow region	0 ^(*)^	.	.	0 ^(*)^	.	.
2.7.	Urban/rural residence						
2.7.1.	Rural area	0.277	0.007	0.000	0.099	0.012	0.000
2.7.2.	Urban area	0 ^(*)^	.	.	0 ^(*)^	.	.
2.8.	Type of injury						
2.8.1.	Fracture of skull and facial bones	1.277	0.022	0.000	0.859	0.090	0.000
2.8.2.	Intracranial injury	0.145	0.025	0.000	1.011	0.087	0.000
2.8.3.	Other Injuries to the head	−0.514	0.024	0.000	−0.088	0.087	0.313
2.8.4.	Injuries to the neck	0.445	0.024	0.000	1.164	0.084	0.000
2.8.5.	Open wound of thorax	0.133	0.021	0.000	1.182	0.081	0.000
2.8.6.	Other injuries to the abdomen, lower back, lumbar spine and pelvis	−0.218	0.026	0.000	1.151	0.085	0.000
2.8.7.	Injuries to the abdomen, lower back, lumbar spine and pelvis	2.272	0.024	0.000	3.650	0.083	0.000
2.8.8.	Injuries to the limbs	1.064	0.021	0.000	2.842	0.079	0.000
2.8.9.	Effects of foreign body entering through natural orifice	2.211	0.054	0.000	3.484	0.111	0.000
2.8.2.	Burns and corrosions	2.648	0.038	0.000	4.202	0.090	0.000
2.8.3.	Frostbite	2.016	0.245	0.000	4.991	0.381	0.000
2.8.4.	Poisoning by narcotics and psychodysleptics [hallucinogens]	6.100	0.058	0.000	9.162	0.118	0.000
2.8.5.	Toxic effect of carbon monoxide	4.422	0.115	0.000	6.240	0.211	0.000
2.8.6.	Other poisoning by drugs, medicaments and biological substances, toxic effects of substances chiefly nonmedicinal as to source	3.540	0.055	0.000	3.914	0.123	0.000
2.8.7.	Hypothermia and other effects of reduced temperature	3.094	0.102	0.000	4.395	0.200	0.000
2.8.8.	Asphyxiation	5.576	0.020	0.000	5.678	0.078	0.000
2.8.9.	Effects of lightning, drowning and nonfatal submersion, vibration, electric current and other specified effects	2.819	0.043	0.000	4.360	0.098	0.000
2.8.10.	Other injury, poisoning and consequences of external causes	0 ^(*)^	.	.	0 ^(*)^	.	.
2.9.	Presence of alcoholic intoxication at death						
2.9.1.	No	0.258	0.023	0.000	0.465	0.039	0.000
2.9.2.	Yes	0 ^(*)^	.	.	0 ^(*)^	.	.
2.10.	Specific location of death						
2.10.1.	Home	0.498	0.008	0.000	0.401	0.014	0.000
2.10.2.	Residential institution	0.316	0.034	0.000	0.140	0.058	0.016
2.10.3.	School, other institution and public administrative area	0.497	0.030	0.000	0.552	0.057	0.000
2.10.4.	Sports and athletics area	−0.916	0.021	0.000	−0.024	0.037	0.504
2.10.5.	Street and highway	0.003	0.050	0.958	−1.065	0.108	0.000
2.10.6.	Trade and service area	0.262	0.041	0.000	−0.266	0.124	0.031
2.10.7.	Other specified places	−0.376	0.014	0.000	−0.272	0.026	0.000
2.10.8.	Unspecified place	0 ^(*)^	.	.	0 ^(*)^	.	.
3.	Homicide	the base outcome					

^(*)^ This parameter is set to zero because it is redundant

## Appendix B

**Table 9:** Distribution of deaths by sex and by cause according to officially registered vital statistics data and our reclassified figures

	Number of deaths (thousand)					SDR per 100,000				
	Total	Non-transport accidents	Suicide	Homicide	Unclassified	Total	Non-transport accidents	Suicide	Homicide	Unclassified
Male										
Actual	2284	1146	451	286	402	276.1	140.1	53.8	33.5	48.8
ECP ≥ 0.50		1248	493	532	11		152.4	58.9	63.3	1.5
ECP ≥ 0.60		1243	492	508	41		151.8	58.7	60.1	5.5
ECP ≥ 0.70		1235	491	454	104		150.8	58.6	53.3	13.3
ECP ≥ 0.75		1230	491	417	147		150.2	58.6	48.8	18.5
ECP ≥ 0.80		1223	489	376	195		149.5	58.4	44.0	24.3
ECP ≥ 0.85		1216	485	338	246		148.6	57.9	39.5	30.2
ECP ≥ 0.90		1208	477	303	296		147.7	56.9	35.5	36.1
After the population level adjustment		1337	515	432	---		163.3	61.6	51.2	---
Female										
Actual	630	336	91	94	110	62.0	33.4	8.6	9.4	10.7
ECP ≥ 0.50		357	111	154	8		35.5	10.5	15.3	0.6
ECP ≥ 0.60		355	109	148	18		35.3	10.3	14.7	1.6
ECP ≥ 0.70		354	105	139	32		35.2	10.0	13.8	3.0
ECP ≥ 0.75		353	102	133	41		35.2	9.7	13.3	3.9
ECP ≥ 0.80		353	99	126	53		35.1	9.3	12.6	5.0
ECP ≥ 0.85		351	95	114	70		35.0	8.9	11.5	6.6
ECP ≥ 0.90		350	92	101	88		34.8	8.6	10.1	8.5
After the population level adjustment		384	109	137	---		38.1	10.3	13.6	---

## Appendix C

**Table 10:** Distributions of cases by independent variables

	Male				Female			
	Non-transport accidents	Suicide	Homicide	Events of undetermined intent	Non-transport accidents	Suicide	Homicide	Events of undetermined intent
Total	100	100	100	100	100	100	100	100
Knowledge of identity								
Identified person	94.20	98.42	93.74	93.07	95.03	98.87	95.31	94.87
Unidentified person	5.80	1.58	6.26	6.93	4.97	1.13	4.69	5.13
Age group								
0–14	2.38	0.63	0.80	1.05	4.34	1.06	2.10	2.27
15–24	6.67	14.54	11.05	8.36	5.07	12.19	10.27	7.22
25–34	14.85	20.36	21.44	17.27	9.18	13.22	16.69	12.07
35–44	20.72	19.88	24.75	20.59	14.57	14.29	19.07	15.13
45–54	27.32	21.13	23.48	25.26	22.73	16.51	19.83	20.06
55–64	15.88	10.53	10.27	14.71	17.48	11.00	11.95	15.04
65 and older	9.85	12.36	5.80	10.38	24.85	31.47	18.51	26.60
Unknown	2.34	0.57	2.41	2.37	1.78	0.26	1.57	1.61
Year of death								
2000–2002	28.95	31.44	33.40	25.14	28.46	29.89	33.55	24.31
2003–2005	30.02	27.33	30.80	27.38	29.69	26.62	30.82	26.83
2006–2011	41.03	41.24	35.80	47.48	41.85	43.49	35.64	48.86
Type of day								
Monday or a day after national holiday	15.11	16.03	15.12	15.36	15.56	15.99	15.30	15.42
Other weekdays	84.89	83.97	84.88	84.64	84.44	84.01	84.70	84.58
Season of year								
December-February	48.31	50.60	50.71	50.24	47.74	50.75	50.10	49.82
June-August	27.67	20.97	24.90	22.09	32.20	19.98	25.73	23.61
Other months	24.02	28.43	24.39	27.67	20.06	29.27	24.17	26.57
Geographic region								
Central Federal District except Moscow	16.71	12.64	11.35	16.54	14.85	11.39	12.57	15.45
North West Federal District	11.32	9.12	9.07	10.44	11.68	9.89	9.30	11.33
South Federal Districtand Stavropol Krai	7.31	8.29	7.35	14.13	6.21	8.23	7.94	13.54
Volga Federal District	22.80	27.99	19.16	23.20	22.81	23.80	21.50	22.19
Urals Federal District	9.18	10.54	10.74	10.36	9.40	11.18	10.71	11.26
Siberian Federal District	17.44	19.63	23.64	13.14	19.52	21.62	22.24	14.62
Far East Federal District	5.36	6.08	8.35	5.77	5.66	6.04	6.82	5.17
Republics of North Caucasian Federal District	1.09	0.58	1.61	0.64	1.03	0.60	0.77	0.43
Moscow and Moscow Region	8.78	5.13	8.73	5.78	8.83	7.26	8.14	6.00
Urban/rural residence								
Rural area	31.42	40.71	27.80	21.08	32.10	35.22	29.13	21.52
Urban area	68.58	59.29	72.20	78.92	67.90	64.78	70.87	78.48
Type of injury								
Fracture of skull and facial bones	1.21	1.76	7.40	5.41	0.79	0.30	6.44	3.85
Intracranial injury	2.00	0.67	8.13	7.09	1.09	0.40	6.71	3.82
Other Injuries to the head	4.59	0.79	19.42	25.29	3.79	0.37	19.22	24.29
Injuries to the neck	0.71	0.88	7.71	1.97	0.38	0.62	9.16	1.64
Open wound of thorax	1.16	1.85	21.63	4.66	1.10	1.13	16.41	4.01
Other injuries to the abdomen, lower back, lumbar spine and pelvis	0.67	0.55	9.57	4.00	0.68	0.53	8.53	4.91
Injuries to the abdomen, lower back, lumbar spine and pelvis	0.59	1.43	1.99	1.58	2.82	1.28	1.30	2.74
Injuries to the limbs	4.08	2.39	12.16	14.18	4.99	4.71	13.34	18.15
Effects of foreign body entering through natural orifice	6.81	0.11	0.15	0.78	6.03	0.15	0.23	0.70
Burns and corrosions	3.48	0.32	0.29	2.09	6.09	0.73	0.48	3.45
Frostbite	0.85	0.00	0.01	0.07	0.84	0.01	0.01	0.09
Poisoning by narcotics and psychodysleptics [hallucinogens]	5.75	1.81	0.06	8.13	6.40	14.18	0.07	12.00
Toxic effect of alcohol	3.27	0.06	0.00	1.72	1.31	0.06	0.00	0.74
Toxic effect of carbon monoxide	25.81	0.08	0.02	4.81	26.30	0.12	0.02	4.31
Other poisoning by drugs, medicaments and biological substances, toxic effects of substances chiefly nonmedicinal as to source	11.87	0.23	0.08	3.51	13.26	0.13	0.13	3.60
Hypothermia and other effects of reduced temperature	13.39	0.03	0.03	0.86	15.07	0.04	0.03	0.88
Asphyxiation	3.65	86.32	4.30	10.55	2.86	74.78	12.62	8.26
Effects of lightning, drowning and nonfatal submersion, vibration, electric current and other specified effects	10.06	0.21	0.22	2.63	6.18	0.37	0.30	2.24
Other injury, poisoning and consequences of external causes	0.07	0.51	6.83	0.66	0.03	0.10	4.99	0.32
Presence of alcoholic intoxication at death								
No	71.70	98.09	98.09	94.25	71.38	98.45	98.47	94.76
Yes	28.30	1.91	1.91	5.75	28.62	1.55	1.53	5.24
Specific location of death								
Home	55.67	78.46	52.63	40.37	62.24	79.78	62.39	46.50
Residential institution	0.64	0.93	0.83	0.46	0.59	0.66	0.82	0.34
School, other institution and public administrative area	1.00	0.51	1.33	1.91	1.01	0.77	0.94	1.90
Sports and athletics area	6.97	1.07	7.47	4.67	6.81	1.13	4.28	4.06
Street and highway	0.52	0.23	0.56	0.40	0.31	0.10	0.64	0.22
Trade and service area	1.41	0.32	0.46	0.66	0.38	0.09	0.24	0.16
Other specified places	13.00	5.17	7.02	8.32	8.61	3.73	5.54	6.44
Unspecified place	20.81	13.31	29.71	43.22	20.05	13.74	25.16	40.38

## Appendix D

### Estimation of the proportion of hidden transport accidents in the EUIs

We applied mlogit to estimate the proportion of hidden transport accidents in the EUIs. The training dataset includes all events with determined intent for the period 2000–2011 divided to two parts: (1) Transport accidents and (2) a combined category of non-transport accidents, suicides, and homicides. We started with the same list of variables as the one used in our main mlogit model for reclassifying EUIs. However, preliminary analysis showed that the variables for day of week, urban/rural residence, and specific location of death did not significantly contribute to differentiating between (1) and (2). The results of the mlogit model are presented below in Table 11. Using bootstrapping, we found that these outcomes are stable. Table 12 shows that the quality of our predictions is remarkably high, with 98.5 % of these cases correctly identified. Since we have to predict only two types of events, (1) vs. (2), all estimated classification probabilities (ECPs) are greater than 0.5. The average classification probabilities are very high, 0.989 for males and 0.992 for females. We were unable to establish a single “main predictor” for transport accidents. Any reduction of the variable list decreased the identification of transport accidents. Finally, we applied the result of this modeling to reclassify EUIs. Results are shown in Table 13. Our estimation procedures suggest that the percentage of hidden transport accidents in EUIs is under 1.5 %, with the mean estimated classification probability about 0.99. In sum, the results of these further analyses provide evidence supportive of our decision not to include transport accidents in our reclassification of externally caused EUIs and to reclassify these EUIs only into non-transport accidents, suicides, and homicides.

**Table 11:** Mlogit outputs for classification of (1) transport accidents versus (2) a combined category of non-transport accidents, suicides, and homicides

		Male			Female		
		B	SE	p-value	B	SE	p-value
1.	Transport accidents						
1.0.	Intercept	1.256	0.093	0.000	1.897	0.214	0.000
1.1.	Knowledge of identity						
1.1.1.	Identified person	0.583	0.050	0.000	0.469	0.094	0.000
1.1.2.	Unidentified person	0 ^(*)^	.	.	0 ^(*)^	.	.
1.2.	Age group						
1.2.1.	0–14	0.908	0.086	0.000	0.572	0.163	0.000
1.2.2.	15–24	0.587	0.076	0.000	0.826	0.155	0.000
1.2.3.	25–34	0.294	0.075	0.000	0.112	0.153	0.466
1.2.4.	35–44	0.068	0.074	0.359	−0.012	0.152	0.939
1.2.5.	45–54	−0.183	0.074	0.014	−0.309	0.152	0.041
1.2.6.	55–64	−0.335	0.075	0.000	−0.441	0.153	0.004
1.2.7.	65 and older	−0.047	0.076	0.538	0.055	0.150	0.713
1.2.8.	Unknown age	0 ^(*)^	.	.	0 ^(*)^	.	.
1.3.	Year of death						
1.3.1.	2000–2002	0.176	0.016	0.000	0.145	0.035	0.000
1.3.2.	2003–2005	0.126	0.017	0.000	0.053	0.035	0.129
1.3.3.	2006–2011	0 ^(*)^	.	.	0 ^(*)^	.	.
1.4.	Season of year						
1.4.1.	December-February	−0.150	0.016	0.000	−0.326	0.035	0.000
1.4.2.	June-August	−0.383	0.019	0.000	−0.513	0.041	0.000
1.4.3.	Other months	0 ^(*)^	.	.	0 ^(*)^	.	.
1.5.	Geographic region						
1.5.1.	Central Federal District	0.065	0.032	0.039	0.035	0.066	0.599
	(except Moscow)						
1.5.2.	North West Federal	0.049	0.034	0.150	−0.047	0.070	0.498
	District						
1.5.3.	South Federal	0.354	0.036	0.000	0.241	0.077	0.002
	District and Stavropol						
	Krai						
1.5.4.	Volga Federal District	−0.670	0.028	0.000	−0.892	0.058	0.000
1.5.5.	Urals Federal District	−0.086	0.034	0.010	−0.404	0.070	0.000
1.5.6.	Siberian Federal District	−0.498	0.029	0.000	−0.489	0.061	0.000
1.5.7.	Far East Federal District	0.845	0.036	0.000	0.800	0.074	0.000
1.5.8.	Republics of North	5.357	0.031	0.000	5.616	0.064	0.000
	Caucasian Federal						
	District						
1.5.9.	Moscow City and	0 ^(*)^	.	.	0 ^(*)^	.	.
	Moscow Region						
1.6.	Type of injury						
1.6.1.	Fracture of skull and	3.258	0.076	0.000	3.472	0.185	0.000
	facial bones						
1.6.2.	Intracranial injury	4.119	0.075	0.000	4.261	0.183	0.000
1.6.3.	Other injuries to the	3.417	0.073	0.000	3.354	0.178	0.000
	Head						
1.6.4.	Injuries to the neck	2.995	0.079	0.000	2.969	0.188	0.000
1.6.5.	Open wound of thorax	3.090	0.074	0.000	3.086	0.181	0.000
1.6.6.	Other injuries to the	3.084	0.079	0.000	3.041	0.188	0.000
	abdomen, lower back,						
	lumbar spine, and pelvis						
1.6.7.	Injuries to the abdomen.	2.731	0.090	0.000	2.473	0.198	0.000
	lower back. lumbar						
	spine and pelvis						
1.6.8.	Injuries to the limbs	3.638	0.073	0.000	3.532	0.176	0.000
1.6.9.	Effects of foreign body	−2.411	0.081	0.000	−3.145	0.198	0.000
	entering through natural						
	orifice						
1.6.10.	Burns and corrosions	0.286	0.088	0.001	−0.423	0.196	0.031
1.6.11.	Frostbite	−4.127	0.225	0.000	−5.088	0.511	0.000
1.6.12.	Poisoning by narcotics	−2.516	0.077	0.000	−3.327	0.185	0.000
	and psychodysleptics						
1.6.13.	Toxic effect of carbon	−7.957	0.088	0.000	−8.373	0.203	0.000
	monoxide						
1.6.14.	Other poisoning by	−2.754	0.082	0.000	−2.767	0.189	0.000
	drugs. medicaments, and						
	biological substances.						
	Toxic effects of						
	substances chiefly						
	nonmedicinal as to						
	source						
1.6.15.	Hypothermia and other	−3.185	0.083	0.000	−3.644	0.191	0.000
	effects of reduced						
	temperature						
1.6.16.	Asphyxiation	−1.036	0.073	0.000	−1.859	0.180	0.000
1.6.17.	Effects of lightning,	−0.907	0.078	0.000	−0.772	0.189	0.000
	drowning, and nonfatal						
	submersion. Vibration.						
	Electric current and						
	Other specified effects.						
1.6.18.	Other injury. Poisoning	0 ^(*)^	.	.	0 ^(*)^	.	.
	and consequences of						
	external causes						
1.7.	Presence of alcoholic intoxication at death						
1.7.1.	No	−9.429	0.021	0.000	−9.931	0.045	0.000
1.7.2.	Yes	0 ^(*)^	.	.	0 ^(*)^	.	.
2.	Non-transport accidents, suicides, and homicides	Base outcome					

(*) This parameter is set to zero because it is redundant

**Table 12:** Distribution of deaths with known causes by actual and predicted cause (per 1000)

	Male				Female			
	Actual cause				Actual cause			
Predicted cause	Transport accidents	Non-transport accidents, suicides, and homicides	Total	Share of agreement	Transport accidents	Non-transport accidents, suicides, and homicides	Total	Share of agreement
All cases								
Transport accidents	147	14	161	0.91	186	9	195	0.95
Non-transport accidents, suicides, and homicides	1	837	839	1.00	1	804	805	1.00
Total	149	851	1000		187	813	1000	
Share of agreement	0.99	0.98		0.98	0.99	0.99		0.99

**Table 13:** Distribution of EUIs by estimated type of events for males and females

	Male		Female	
	Percentage	Mean EPC	Percentage	Mean EPC
Transport accidents	1.5	0.86	1.2	0.90
Non-transport accidents, suicides, and homicides	98.5	0.99	98.8	0.99

## Appendix E

### Alternative redistributions of Events of Undetermined Intent (EUIs)

This Appendix shows the redistributions of EUIs via two simple alternatives relative to the model-based redistributions we provide in the text. The first is the default method of redistribution. This is based on the assumption that for every sex and age group the shares of non-transport accidents, suicides, and homicides among all EUIs are exactly the same as the shares of these three causes in the total number of officially registered total number of non-transport accidents, suicides, and homicides (i.e., non-transport accidents + suicides + homicides). In the tables we label this “default redistribution.” The second method is based on the assumption that each EUI should be divided into shares equal to the three estimated classification probabilities referring to non-transport accidents, suicides, and homicides. Under this hypothesis we calculated the mean number of hidden events of each type for all sex and age groups of EUIs. In the tables we label this proportional share approach as “sharing-based redistribution.” Table 14 provides this information when we included deaths from transport accidents in the calculation (see discussion in text and Appendix D) and Table 15 provides this information when we excluded deaths from transport accidents.

**Table 14:** Distributions of all deaths, including transport accidents, from external causes before and after redistributions of events of undetermined intent

	Deaths (in thousands)				Standardized Death Rate per 100,000			
	Vital statistics	Model-based redistribution	Default redistribution	Sharing-based redistribution	Vital statistics	Model-based redistribution	Default redistribution	Sharing-based redistribution
Total	2913	2913	2913	2913	160.1	160.1	160.1	160.1
Non-transport accidents	1481	1722	1797	1656	82.2	95.3	101.3	91.9
Suicides	541	624	656	631	29.1	33.7	34.6	33.9
Homicides	379	568	460	626	20.7	31.0	24.2	34.3
EUIs	512	0	0	0	28.0	0	0	0

**Table 15:** Distributions of deaths, not including transport accidents, from external causes before and after redistributions of events of undetermined intent

	Deaths (in thousands)			Standardized Death Rate per 100,000		
	Model-based redistribution	Default redistribution	Sharing-based redistribution	Model-based redistribution	Default redistribution	Sharing-based redistribution
All EUIs	512	512	512	28.0	28.0	28.0
Non-transport accidents	240	317	175	13.1	19.1	9.6
Suicides	83	115	90	4.6	5.5	4.8
Homicides	189	81	248	10.3	3.5	13.6
